# Oral microbiome and health

**DOI:** 10.3934/microbiol.2018.1.42

**Published:** 2018-01-12

**Authors:** Neetu Sharma, Sonu Bhatia, Abhinashi Singh Sodhi, Navneet Batra

**Affiliations:** 1 Department of Microbiology, GGDSD College, Sector 32 C Chandigarh, India; 2 Department of Biotechnology, GGDSD College, Sector 32 C Chandigarh, India

**Keywords:** microbiome, oral cavity, HOMD, biofilm, periodontitis

## Abstract

The oral microbiome is diverse in its composition due to continuous contact of oral cavity with the external environment. Temperatures, diet, pH, feeding habits are important factors that contribute in the establishment of oral microbiome. Both culture dependent and culture independent approaches have been employed in the analysis of oral microbiome. Gene-based methods like PCR amplification techniques, random amplicon cloning, PCR-RELP, T-RELP, DGGE and DNA microarray analysis have been applied to increase oral microbiome related knowledge. Studies revealed that microbes from the phyla *Firmicutes, Proteobacteria, Bacteroidetes, Actinobacteria, Fusobacteria, Neisseria*, TM7 predominately inhabits the oral cavity. Culture-independent molecular techniques revealed the presence of genera *Megasphaera*, *Parvimonas* and *Desulfobulbus* in periodontal disease. Bacteria, fungi and protozoa colonize themselves on various surfaces in oral cavity. Microbial biofilms are formed on the buccal mucosa, dorsum of the tongue, tooth surfaces and gingival sulcus. Various studies demonstrate relationship between unbalanced microflora and development of diseases like tooth caries, periodontal diseases, type 2 diabetes, circulatory system related diseases etc. Transcriptome-based remodelling of microbial metabolism in health and disease associated states has been well reported. Human diets and habitat can trigger virus activation and influence phage members of oral microbiome. As it is said, “Mouth, is the gateway to the total body wellness, thus oral microbiome influences overall health of an individual”.

## 1. Introduction

The microorganisms are present on surface tissues of all human beings such as skin, oral cavity, respiratory tract, gastrointestinal tract and urogenital tract. The number and type of these microbes varies with age, diet and personal hygiene levels of a person. They are collectively referred as normal microflora of human body. The diversity of indigenous microflora is mainly driven by multiple factors including environmental factors, diet of individuals, migrations and genetic factors [1]. With continuous advancement in discovery of new drugs and progress in medical fields some of the populations of native microflorais getting disappeared [2]. Oral and stool communities were reported to be most diverse type in terms of microbial diversity [3]. The changes in microecology directly influences person's microbiota due to exposure to different factors such as antibiotics, vaccines, active or passive smoking etc., which varies from person to person and hence produce variable effects [4]. One such example of diminishing microbiota is disappearance of *Helicobacter pylori* from human population due to changes in factors discussed above. The studies reported that presently this bacterium is present in less than ten percent of children in USA in their stomach. This bacterium plays an important role in modulating immunological, endocrine and various physiological responses in the stomach [5]. Some strains are reported to play important role in preventing gastroesophageal reflux diseases (GERD) as it influences the secretion of gastric acid in stomach [2]. The disappearance of strains leads to increase in incidence of GERD in populations from where this bacterium had diminished.

The normal microflora of oral cavity mainly comprises of those microbes which have the ability to stick to the surfaces like gums and teeth and thus resist their removal. However, non-adherent microbes are removed by mechanical flushing of the mouth as a result of tongue's movement during chewing and talking into the stomach and hence destroyed there [6]. The easy availability of epithelial debris as nutrients, water and suitable temperature and pH aids in colonization of microbiota in oral cavity. The different sites invaded by different microbes in oral cavity in the form of biofilms. At the time of birth oral cavity of humans is microbe free and is colonized by microbes within hours of their birth from surrounding environment. However, it is difficult to define exact composition of oral microbiome because mouth has exposure to exogenous bacteria in food, water and air. Social contact and kissing can also result in changes in microbial community [7]. The populations of invading microbes initially comprises mostly of aerobes and obligate anaerobes related mostly to the genera *Streptococcus, Actinomyces, Veilionella, Neisseria* and some yeasts. Later on after the eruption of teeth, anaerobic forms viz. *Prevotella, Fusarium* etc. dominate due to presence of anaerobic environment between gums and teeth. *Streptococccus* spp. such as *S. parasanguis* and *S. mutans* grows on enamel and some colonize gingival epithelial surfaces and saliva [8]. The production of various adherence factors facilitates their attachment and colonization.

## 2. Oral environment and indigenous microflora

Conditions in oral cavity such as physical and chemical parameters keeps on changing due to continuous exposure to external environment. The microflora inhabits different sites in oral cavity such as saliva, teeth, tongue, cheek, hard and soft palate, gingival sulcus and lips. Though saliva is believed to have vast number of bacteria, but poor nutrient availability and variable flow rate would be a factor of debate of the survival of native microbiota in saliva. To a large extent, the organisms found in the saliva are those shed by or dislodged from other oral surfaces, in particular the dorsal surface of the tongue [9]. The earlier studies reported that the organisms inhabiting saliva known as planktonic organisms which represent over 99.9% of all bacteria present in the oral cavity. These organisms played a vital role in constructing optimal environment for their survival by better absorption of nutrients and interaction with other species through quorum sensing to regulate their growth rates [10]. The oral health of an individual depends on the presence of healthy biofilm of indigenous microflora on surface of gums, teeth and linings of oral cavity. The inhabitant organisms would have less probability of surviving in the environment which is pathological to the hosts. This type of situation where resident microbes loses homeostasis leads to onset of several oral diseases [11]. The proposed mechanism behind this condition is attributed to pleomorphism, the natural phenomenon observed in microbes by which they altered themselves from friendly to a pathogenic state in response to adverse environmental conditions or consumption of antimicrobial agents [12]. The oral cavity further act as a major gateway to pharynx, tonsils, lungs, eustachian tubes, middle ear etc. and thus has a substantial prospect of invading these sites [13]. Many studies were reported on direct influence of oral microbiome with some prominent systemic diseases such as cardiovascular diseases [14], preterm birth [15], diabetes [16], stroke [17] and pneumonia [18]. The host produces variety of antimicrobial compounds and enzymes like lysozyme, amylases, immunoglobulins A, G, M, mucus layers and shedding of epithelial cells. These all factors presented a barrier for the establishment of new microflora in the mouth. A part from these enduring bacterial species also produced different class of bacteriocins which further strengthened this barrier. The principal source of nutrition for indigeneous microbes is saliva, food consumed by host and variety of byproducts produced by interspecies which supports their growth and multiplication. Due to continuous variable physico-chemical conditions in oral cavity, there exists a constant evolution of adaptive traits among the microflora. Mostly such traits were carried by transposons and hence were further transmitted to other species present there [6].

## 3. Bacteria and their interactions in oral cavity

**Figure 1.**
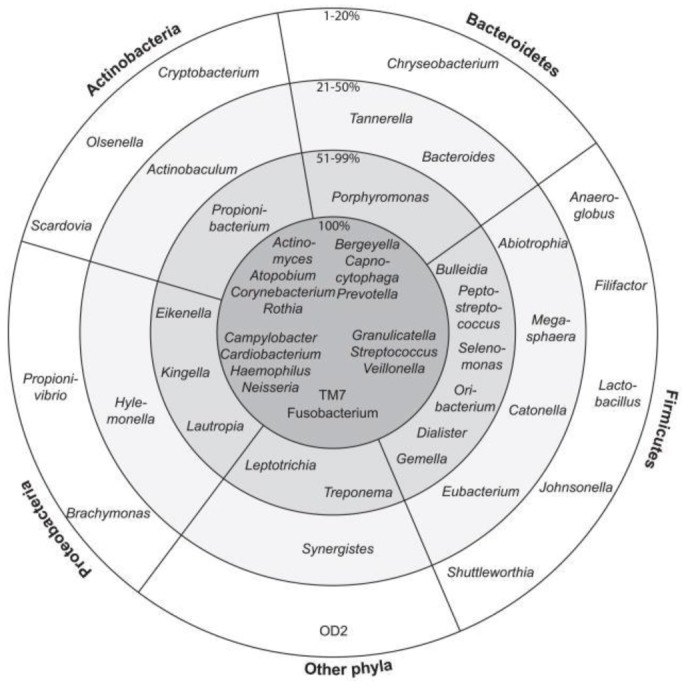
Schematic representation of oral community (Genera grouped according to phylum) among 10 healthy individuals [46]. Inner circle: Bacterial genera found in all 10 individuals (100%); Second circle: Present in 6–9 out of 10 individuals (51–99%); Third circle: Present in 3–5 individuals (21–50%); Outer circle: Present in 1–2 individuals (1–20%).

Antony van Leeuwenhoek was the first one to report the findings on bacteria from the sample derived from his oral cavity in late sixteenth century [19]. The findings of Leeuwenhoek paved the way for the beginning of exploration of more microbes other than bacteria. After the colon, the mouth was reported to have highly complex bacterial communities [3]. The discovery of culture media by Robert Koch and his associates helped in preliminary identification of oral microbiome based on their nutritional and oxygen requirements thus leading to identification of obligate/facultative aerobes and anaerobes which played crucial role in certain dental problems. These studies further helped in identification of certain antimicrobials for curing these problems [20]. But till date most of the microflora of mouth is under investigation as most of the species are unculturable and genomic similarities didn't help in determination of organisms as these were based on comparison of short sequence length [21]. The other problems associated with oral microbiome studies were low concentration of certain bacterial species, reproduction of oral environment in laboratory conditions and failure to mimic nutritional requirements. With the advancement in molecular tools for identification of microbial species, diverse unculturable flora are identified. The molecular techniques which were employed earlier, mainly comprised of PCR amplification and 16S rRNA gene sequencing. These techniques failed in detection of many bacterial species [22]. With the sequencing of human genome, it was believed that it would act as a boon for studying diseases and other factors relating to humans but didn't turn out to be as helpful as was limited to impart knowledge just about the genetic composition [23]. The major outbreak in this field was achieved via introduction of Human Microbiome Project which was aimed at identification of important commensal species responsible for healthy human microbiome [23],[24]. The most promising step in determining the actual status of human microflora was achieved with the advancement of metagenomics where total DNA of microbial community is obtained and studied; hence eliminating the need of culture based studies and PCR amplification. The progress made in field of next generation sequencing further improved the scope of studies related to human microflora [25],[26]. The inhabitation of any site in human body by microflora depends on its adaptability to the environment of that site or due to their drift to the neighboring tissues. The later phenomenon is known as normal distribution [27]. Mostly the associations of normal microbiota with human tissues are either mutual or symbiotic. One such example is prevalent forms of few commensal bacteria. They prevents pathogenic forms to locate themselves at the corresponding sites as the later has to infringe the barrier established by the former [28], but they may turn out to be parasitic due to some factors viz., environmental or other factors such as diseased state, low immunity levels resulting in successful establishment of pathogen that leads to infectious state. Syntrophic association was reported between species of *Veillonella* and *Streptococci* where former species fed on lactate produced by the later [29]. Synergistic association of multiple species was observed in case of dental caries, the most common dental problem among humans after periodontitis [30]. Important interactions found in biofilms, which consists of communities of different microbes and their function and survival, primarily depends upon complex symbiotic interactions among members of biofilms [31]. Quorum sensing (QS) has been widely used by many bacterial and fungal species especially to regulate biofilm development and maintenance [32]. Some of the examples includes peptide auto inducers, auto inducers-2 (AI-2), acyl homoserine lactones and fungal QS systems [33]. The peptide auto inducers are the main QS system associated with oral cavity. Such associations were reported to play major role in dental plaques where more than 700 species were identified [34],[35]. Dental biofilms were studied and reported for different associations like coaggregation which helps in adhesion of species to tooth surfaces and metabolic cooperation through interlinked food chains of various species [34],[36]. Bacteriocin production by different members of biofilms community accounts for biodiversity and ecological suitability of microbes. AI-2 (Autoinducer) pathway is used by *Streptococcus gordonii* and *Porphyromonas gingivalis* while growing as biofilm in oral cavity [37]. Different species of natural inhabitant of oral cavity *Streoptocoocus* such as *S. mutans, S. gordanii* and *S. mitis* produced bactericions through quorum sensing and thus regulate the formation of biofilms [38],[39]. Such interactions played an important role in community interactions within biofilms and helped in coexistence of different species in similar oral environment [40],[41]. *S. gordanii* was believed to play role in minimizing dental plaques due to its ability to produce hydrogen peroxide which is fatal for growth of invading bacteria. The interactions found among different members of oral biofilm were found to be very complex and may be antagonistic or synergestic. The other important species associated with biofilms was *Actinomyces naeslundii* which removed hydrogen peroxide and aid in growth of *S. gordonii* in absence of arginine. On the other hand hydrogen peroxide produced by *S. gordonii* is fatal for growth of *A*. *naeslundii* [42]. The other such interactions were also reported for *Fusobacterium nucleatum* with *Streptococcus cristatus* where former helped lateral in successful establishment in epithelial cells [43]. However, *S. cristatus* attenuates *F. nucleatum* driven cytokine expression in epitheleial cells [44]. Apart from extracellular biofilms, large communities of intracellular bacteria were also associated with gingival and buccal epithelial cells of oral cavity [23]. Most of the intracellular communities found in mouths of healthy individuals believed to have pathogenic properties but didn't show any symptoms due to evolution of tolerance by the host [45]. Many such associations were reported among various species. The study of such interactions would be very helpful in designing strategies for prevention, diagnosis and cure of some commonly occurring oral diseases (Figure 1) [46].

## 4. Non bacterial members of oral cavity

The most diverse forms of microbes were reported in oral cavity such as protozoa, fungi and viruses [23]. The protozoan population is believed to be mainly saprophytic and most commonly reported members were *Entamoeba gingivalis* and *Trichomonas tenax*. *E. gingivalis* was reported to be present in higher numbers at periodontally diseased sites indicating some associations with other members found at those sites [47]. Among fungus, *Candida* species is the most prevalent form associated with oral cavity and is primarily responsible for different oral infections. *Candida albicans* is the normal habitant of oral cavity and was reported to form biofilms on solid surfaces [48]. Once successfully grown on solid surfaces, biofilms of *C. albicans* can invade the adjoining cells. Ghannoum et al. [49] reported 85 fungal genera in culture independent studies carried out in 20 healthy hosts and the main species observed were those belonging to *Candida, Cladosporium, Aureobasidium, Saccharomycetales, Aspergillus, Fusarium* and *Cryptococcus*. But further studies are required to validate the presence of the these unusual genera in mouth that whether they are transient or permanent members of normal microbiota. Viruses were found both in active and latent forms and responsible for wide range of diseases of oral cavity and its associated organs. According to a study by Wang et al. [50] several phages has been identified from salivary and dental plaque samples including *Corneybacterium* phage, *Lactococcus* phage, *Pseudomonas* phage etc., belonging to the family of *Siphoviridae*, *Myoviridae* and *Podoviridae* respectively [50]. It has been reported that phages are quite stable in the oral cavity and this can be correlated to the survival of the phages by inactivation of the bacterial defence mechanisms which leads to their establishment in the oral cavity [51]. Analyzing several bacteriophage communities, it was found that the presence of bacteriophages in the oral cavity is highly individual and gender specific when compared with phages from other habitats inside the human body like gut and skin etc. [52]. Phages in the oral cavity are also associated with several diseases like endocarditis due to presence of high number of virulence genes [53]. Ly et al. [54] reported the role of certain viruses in imparting pathogenic characteristics to their host bacteria which were normal inhabitants of oral microbiome. Hence their role as active participants cannot be ignored. The most common viral borne infections of salivary glands are mumps and rabies. The other common invading viral strain is *Herpes simplex*, which is responsible for varied clinical symptoms at different age levels. The virus is quite lethal in action as have the capability to escape host immune response and enter latent phase. The virus easily gets triggered at later stage in response to stress or exposure to ultraviolet radiations and mainly associated with development of *Herpes labialis* commonly known as cold sores in the carrier [55]. Herpes viruses were also associated with periodontitis along with bacteria [56]. *P. gingivalis*, commonly found in oral cavity had reported to increase the CCR5 expression, an important receptor for inducing infections by some HIV strains and hence may have some role in development of AIDS [57],[58]. Human papilloma virus (HPV) had been associated with several oral disorders including condylomas, papillomas and epithelial hyperplasia and some non oral disorders like head and neck squamous cell carcinoma [59],[60]. Oral virome studies carried by Pride et al. [61] reported the prevalence of bacteriophages in oral cavities. Another study by Abeles et al. [62] reported that phage community remain stable and specific with respect to single individual. In patients with periodontal disorders viral communities remain homogeneous as compared to healthy individuals [54]. Several *in vitro* and metagenomic studies reported abundance of bacteriophages, the main factors of pathogenecity in mouth where they lysogenize some disease causing pathogens like *Enterococcus faecalis* and *Aggregatibacter actinomycetemcomitans* [63],[64] and thus induced several dental disorders. Some of the species of methanogens belonging to Archaea were also found in oral microbiota. The most studied were *Methanobrevibacter oralis*, *Methanobacterium curvum/congolense* and *Methanosarcina mazeii*. Like Protozoan members, archaeal population was also found to be raised in people suffering from periodontitis [65],[66].

## 5. Gene transfer among oral microbiota

The microbiota of mouth continually faces variable physical, chemical and biological pressures due to its direct contact with external environment. The oral microbiota thus makes several adjustments for their survival in this variable background at their metabolic level which is accompanied by many changes observed at genetic level. Horizontal gene transfer (HGT) was observed as an important mechanism in evolution of *Streptococcus* genus having maximum abundance in oral cavity for acquiring adaptive traits to survive in complex environment [67]. These observations were supported by studies carried on factors influencing HGT which reported occurrence of this phenomenon in bacterial population having genes similarity of more than 99% at nucleotide levels. Thus different environments with variable components will have their own metagenome as per adaptations made to local stress factors and type of interactions among members of that habitat [68]. Although HGT is carried out by conjugation, transduction and transformation but new mechanism was observed in biofilms of oral microbiota to attain new characters. Most of the population of Gram positive and some Gram negative bacteria inhabiting oral cavity was observed to release membrane vesicles containing polysaccharides, proteins, DNA, plasmid borne antibiotic resistance genes and virulence genes [69]. The released DNA and other components were reported to be utilized as successful substrates to transform other members of community. Mashburn-Warren and Whitley [70] reported one such vesicle borne transformation from *E. coli* to *Salmonella*. For the treatment of periodontitis, doxycycline resistance encoding genes were successfully reported by Warburton et al. [71]. The other examples of HGT in oral microbiota includes transfer of DNA from transient species to permanent inhabitants as was observed in metagenomic libraries of DNA isolated from saliva of different individuals [72]. The transfer of certain antibiotic resistance characters present on mobile genetic elements (MGEs) was reported by Ciric et al. [73]. One such example is transposon Tn916 which carries several resistance genes and acts as prime factor for transfer of these characters to other members in oral cavity [74]. The other less studied phenomenon responsible for transfer of traits of oral microbiome is via vertical transmission. As reported in case of population of *Helicobacter pylori* which varies among different individuals depending on their diet, migration, genetic isolation etc., in the same way it would be advantageous if some bacterial species can be identified in oral cavity too. The study of diversity of such bacterial species among different communities would help to study factors influencing evolutionary adaptations among members of oral community. However, the most important condition associated is that species should reveal high levels of vertical transmission [75]. Few evidences supporting this phenomenon is based on the studies carried out in case of mother-child and twins [76],[77]. Bacteriophages are also responsible for transferring certain gene functions to the bacteria by horizontal gene transfer mechanism [62],[78]. For instance, *Siphoviridae* follows lysogenic life cycle and therefore shows high possibility of gene transfer through horizontal gene transfer mechanism specifically in the oral cavity. It has been reported that phages actively transfer their genes to the bacterial community by establishing themselves inside the bacterial cell as prophages [51]. In contrast many lytic phages like *Mycoviridae* are responsible for maintaining a dynamic equilibrium between different bacterial species for host by frequently eliminating their hosts [51],[79]. The studies based on vertical transmission are still in its infancy stage and requires further findings to support this fact. Such studies would also be helpful in studying complex interaction occurring in mouth among different members of the cavity and the factors responsible for dominance of certain type of species in that environment.

## 6. Influence of external factors on oral microbiome

Human oral microbiota show little variation in its composition with variable geographical location [75]. Microbial community homeostasis is influenced by many factors like interactions with external environment, community associations and internal interactions between different organs etc. Microbiota can be acquired from mother to child at the time of birth by vertical transmission. Mode of delivery of the new born determines types of microorganisms encountered first. Microorganisms can be derived from vagina in case of normal delivery or skin in case of caesarean [80]. Studies showed that vaginally born infants at the age of 3 months, have greater taxonomic diversity of oral microbiome [81]. Infant's oral microbiome can also be affected by feeding habits. Studies has suggested that oral cavity of breast-fed infants possess oral lactobacilli with antimicrobial properties which was lacking in instant formula-fed infants [82],[83]. Oral microbiome diversity can also be acquired by horizontal transmission among people by sharing of similar environment and habitats [84],[85]. As in case of certain traits which are attained by individuals during their association with environment like adaptive immunity, micobiome is also influenced by such factors which have not been given much emphasis while carrying out the studies associated with their diversity and characterization. The effect of vaccination having attenuated microbes, health status of a person, age and effects of certain habits which vary from person to person. Smoking directly influences the composition of the oral community. Both active and passive smoking exposure effects colonization of upper airways by pathogenic species as reported by Brook and Grober [86]. Smoking influence colonization of pathogenic microbes by impairing host immune response [87], disruption of nasal mucociliary passage and by enhancing their binding to epithelial cells [88],[89]. Apart from this many pathogens were reported to be present in cigarette like *Acinetobacter, Bacillus, Burkholedria, Clostridium, Klebsiella, Pseudomonas aerugionosa* and *Serratia lineages* and hence can be a source of diseases caused by them [90]. The members of indigeneos microbiome comprising of *Prevotella* and *Peptostreptococcus* which prevents growth of invading pathogens were reported to be absent in smokers and presence of certain pathogens like *Haemphilus influenza, Streptococcus pneumonia* and *Moraxella catarrhalis* were documented in nasopharynx of smokers [91]. Certain species responsible for periodontitis like *Fusobacterium, Parvimonas, Campylobacter* and *Bacteroides* were present in subginigival environment of oral cavity of cigarette smokers [92]. It has been observed that after smoking is ceased, nasopharynx and oral cavity is again dominated by its friendly indigenous microflora [91],[93]. Some reports based on recent techniques like univariate analysis and machine learning approach indicated the increase in number of *Megasphaera spp.*, *Firmicutes, Streptococcus, Vellionella* and *Atopobium spp., Eggerthella, Erysipelotrichaceae I.S., Dorea, Anaerovorax* and *Eubacterium spp.* in smokers as compared to non smokers. Most of the species mentioned above are mainly associated with oral infections. The only species whose number was found to reduce in nasopharynx of smokers is *Shigella spp.* [4]. The significant alterations were observed in indigenous microbial communities of oral cavity and nasopharynx of smokers and hence can be linked to the incidence of more infections in people linked with smoking habits as compared to non smokers. Further studies are required to observe the diversity of microflora of passive smokers by using recent culture independent techniques so as to have complete profile of oral microflora which have not been traced till date. The other important factor contributing the change in nasopharyngeal community is use of vaccines. The structure of community also varied with diseased status of individual. There was a variation in microbial communities in individuals having HIV infection as compared to non-infected children [94]. The decrease in concentration of antibodies required to prevent colonization of *Pneumococci* organism was observed even after three doses of pneumococcal conjugate vaccine (PCV), hence depicting the failure of effectiveness of vaccine in absence of its booster dose. HIV infected children were found to be more prone to colonization by pneumococci by vaccine serotypes as compared to uninfected due to decrease in mucosal and systemic immunity. The colonization by pneumococci further effects invasion by disease causing pathogens including *S.aureus* and *H.influenzae* in HIV-uninfected children. The negative associations between *S. pneumonia* and *S. aureus* observed in uninfected children were due to the production of hydrogen peroxide by the former which is lethal for the colonization of lateral species. Similar associations were observed between *S. aureus* and *H. influenza* in uninfected children. The development of adaptive immunity against *pneumococci* in older age after pneumococcal colonization at early stage and presence of negative association in un-infected children was attributed not only to bacterial associations but also depends on host's immunological response which has been weakened in HIV infected hosts [94]–[96].

## 7. Oral microbiome and disease

### 7.1. Periodontal diseases

The colonization of periodontal pocket formed as a result of loss of attachment between teeth and gingivae by anaerobic bacteria results in development of periodontitis [97]. The other type of periodontal disease associated with dental plaques is categorized into two categories: chronic and aggressive. The later type had a rapid spread rate and more severe symptoms as compared to former type [98]. But sometimes symptoms are similar to other oral problems therefore early and accurate diagnosis is required to prevent its onset. *Treponema denticola, Porphyromonas gingivalis*, *Tannerella forsythia*, and *Actinobacillus actinomycetemcomitans* are some of the species reported by culture based studies while *Eubacterium saphenum, Filifactoralocis, Anaeroglobus germinates* and *Prevotella denticolla* are some of the species reported by culture independent studies having role in periodontitis [99],[100]. Gingivitis is another common periodontal disease. The disease is accompanied by prevalence of swollen gums due to colonization by Gram negative bacteria such as *Treponema, Fusobacterium* and anaerobic bacterial species viz., *Streptococcus* and *Actinomyces* [101],[102].

### 7.2. Dental caries and endodontic infections

The excess uptake of carbohydrates leads to acid production due to fermentation of carbohydrates by many oral cavity inhabitants which disturbs the buffering capacity of saliva and hence results in tooth decay or dental caries. Bacteria present in oral biofilms can be good or bad depending upon its role in the oral cavity [103]. In a condition of tooth decay there is enamel loss which is promoted by microbial shift in the biofilms from good to bad. In tooth decay demineralization of mineral hydroxyapatite (HAP) crystals occur due to formation of biofilms containing acidogenic and aciduric species. Diet plays an important role in dental caries as supported by a study where *Streptococcus mutans* given excess of glucose will produce lactic acid under aerobic conditions whereas under anerobic conditions lactate, formate, acetate and ethanol are formed [104]. This type of environment also favours the growth of acidophilic bacteria in the oral cavity. The members of genera *Propionobacterium, Bifidobacterium*, *Scarvidia* along with *Streptococccus mutans* and lactobacilli were mainly associated with formation of dental caries [105]–[108]. Certain members were known to produce alkaline products like ammonia which can counter balance the acid produced by fermentation [109]. The complex interactions exist among different members of oral microbiome and strains belonging to different species functions differently under different conditions. Hence more studies were required at strain levels to monitor their activity under different environmental conditions and associations and this may help in controlling the dental caries by modulating the oral environment. Enterococci are believed to be the main causative agents for endodontic infections. It has been observed that they would not be able to compete with normal microbiota of oral cavity but once they reached the root canal, they successfully colonize there and can act as source of many secondary infections. The main source of entercocci is believed to be fermented dairy products like cheese which can harbor such microbes and hence consumption of such products must be avoided by patients undergoing root canal treatments [110],[111]. The use of contaminated instruments may also act as a source of enterococci in the oral cavity. The use of sterilized instruments and avoiding foods which may act as carrier of these potent pathogens may help in controlling such infections.

### 7.3. Role of oral microbiota in non oral disesases

Although good oral hygiene is believed to be the key to healthy oral cavity but it has been observed that sometimes people with sufficient oral hygiene still prone to different oral infections. The prevalence of above symptoms may be due to imbalance in normal microbiota of oral cavity and varied immune response among different individuals. As mentioned earlier oral cavity acts as a gateway to different organs of the body and hence acts as reservoir of different diseases associated with different organs. Raghavendran et al. [112] reported association of development of various types of lung diseases and periodontal disease. *Escherichia coli, Pseudomonas aeruginosa* and *Staphylococcus aureus* were found to colonize the teeth of hospitalized patients [113]. The development of pneumonia in intensive care units patients is most common secondary infection. Hospital acquired pneumonia is divided into two categories: Ventilator-associated pneumonia (VAP) and non ventilallator associated pneumonia [114]. The pathogens inhabiting oral biofilms may get released into oral secretions and effect patients on ventilators in intensive care units [115]. Aspiration pneumonia is another subtype which develops due to inhalation of pathogenic bacteria colonizing the oropharyngeal biofilms [116]. The incidence of this disease would be lower in patients following good oral hygiene practices and patients without teeth [117]. Frequent dental visits may sometimes lead to spread of pathogens to blood stream and from there to different sites like lungs, liver and brain [21],[48]. The onset of cardiovascular disease is associated with oral microbiota. Increment in number of markers of cardiovascular disease such as C-reactive protein, fibrinogen and cellular adhesion protein has been observed in people preaching poor oral hygiene practice [117]. The role of poor periodontal dental health in development of diabetes and complications associated with type 1 and type 2 diabetes has been reported by Borgnakke et al. [118]. The development of systemic inflammation due to periodontal disease has been found to be associated with resistance towards insulin and hence lead to development of diabetes and associated complications. The gut microbiome is directly influenced by oral microbiota due to continuity of oral cavity with gut. Segata et al. [119] reported 45 percent homology of oral and gut microbiome. The presence of *Helicobacter pylori* in dental plaques was directly associated with *H. pylori* related gastric infection [120]. Hence presence of pathogens in oral biofilms may lead to colon related disorders. Some types of cancer are also said to be associated with changes in microbiota [121]. As suggested, *Porphyromonas gingivalis* may trigger in the occurrence of orodigestive cancers, oral squamous cell carcinoma (OSCC) that is most correlated with the oral bacterium [122]. The recent findings also linked association of poor oral health with other diseases like non oral cancers, Rheumatoid arthritis, Alzeheimer's disease and dermentia and pregnancy related complications [123]–[126].

## 8. Identification and characterization of oral microbiome

Oral cavity can be divided into three habitats colonising similar communities. These are the gingival and hard palate, tongue and throat and dental plaque [119],[127]. The compositional analysis of oral microbiome shows that it is relatively stable over time [128]. Sampling from seven different surfaces in mouth, shows colonization of three distinct bacterial communities. Similar microorganisms were found in the buccal mucosa, gingivae and hard palate, another similar group of microbes were found in sites such as tongue, saliva, tonsils and throat (Table 1). Supra and sub gingival plaque show different microbiota [129]. In terms of species richness oral microbiota ranks second after colon [128].

**Table 1.** The core bacterial groups associated with oral cavity.

Sample location	Major Bacterial Groups	References
Buccal mucosa	*Atopobium, Bacilli, Catonella, Pasteurellaceae Prevotellaceae, Streptococcus, Acidobacteriaceae, Xylanibacter, Phocoenobacter, Bacteroidetes, Firmicutes, Proteobacteria, Actinobacteria*	[132]–[137]
Tongue	*Actinomycetales, Bacilli, Fusobacterium, Lactobacillales, Prevotella, Pasteurellaceae, Peptostreptococcus, Streptococcus, Veillonella, Treponema, Synergistes, Clostridiales, Firmicutes, Proteobacteria, Bacteroidetes, Actinobacteria, Chlorobi, T. denticola, T. forsythia, P. endodontalis*,	[138]–[140]
Saliva	*N. flavescens, R. mucilaginosa, S. salivarius, Prevotella histicola, Veillonella parvula, Veillonella atypica, S. salivarius, Streptococcus parasanguinis, Actinomycetales, Fusobacterium, Neisseria, Pasteurellaceae, Prevotella, Streptococcus, Tannerella, Veillonella*	[75],[141],[142]
Hard palate	*Mogibacterium, Catonella, Gemella, Prevotella, Streptococcus*	[136],[143]–[145]
Supergingival and subginival plaques	*Betaproteobacteria, Corynebacterium, Capnocytophaga, Corynebacterium, Firmicutes, Fusobacterium, Neisseriaceae, Pasteurellacea, Prevotella, Streptococcus, Granulicatella, Porphyromonas, Actinomyces, Neisseria, Treponema, Denticola, Tannerella forsythia, E. faecium*	[146]–[148]
Throat	*Actinomyces, Firmicutes, Fusobacterium, Pasteurellaceae, Streptococcus*	[136],[143],[145],[149],[150]
Tonsils	*Firmicutes, Fusobacterium, Mogibacterium, Pasteurellaceae, Prevotella, Streptococcus*	[136],[145],[149]–[151]

As it is said “everything is everywhere, but, the environment selects” [130] to characterize health of oral cavity, oral microbiome must be ruled by a core microbiome which would be helping in maintaince of functional stability and homeostasis in a healthy individual [127]. Oral bacteria show slow growth with critical nutritional parameters and oxygen sensitivity. Bacterial community analysis can be done by culture dependent and culture independent analysis. Novel taxa can be isolated using anaerobic mode of cultivation of oral microbes [131]. The oropharyngeal cavity show quite complex microbial associations. Varieties of microenvironments are associated with oropharyngeal tract comprising of about 700 species of aerobic and anaerobic microbes forming biofilms. Culture based method was used in characterization of dental plaque. In initial stages of plaque formation *Streptococcus spp*. were in abundant with a representation of greater than 50% in all cultivable flora. Presence of *Neisseria spp*. and *Veillonella spp.* was also detected in initial stages of colonization. With maturation of the biofilm, Gram-negative filamentous bacteria dominated [99]. As discussed earlier, culture dependent approach poses some limitations in characterization of bacterial species as all species cannot be readily grown under laboratory conditions. Thus, molecular techniques targeting 16S rRNA gene for identification of microbial communities have been extensively used.

Next generation sequencing techniques like 454 (Roche) pyrosequencing platforms and Illumina sequencing has magnificently enhanced community profiling. 454 pyrosequencing generates sequences of greater than 400 bp lengths which helps in the generation of good phylogenetic tree. A study defining the healthy oral microbiome using 454 pyrosequencing concluded that an individual healthy oral cavity contains over 3600 unique sequences and more than 500 different OTUs (species-level phylotypes) and 88–104 higher taxa [127].

**Figure 2.**
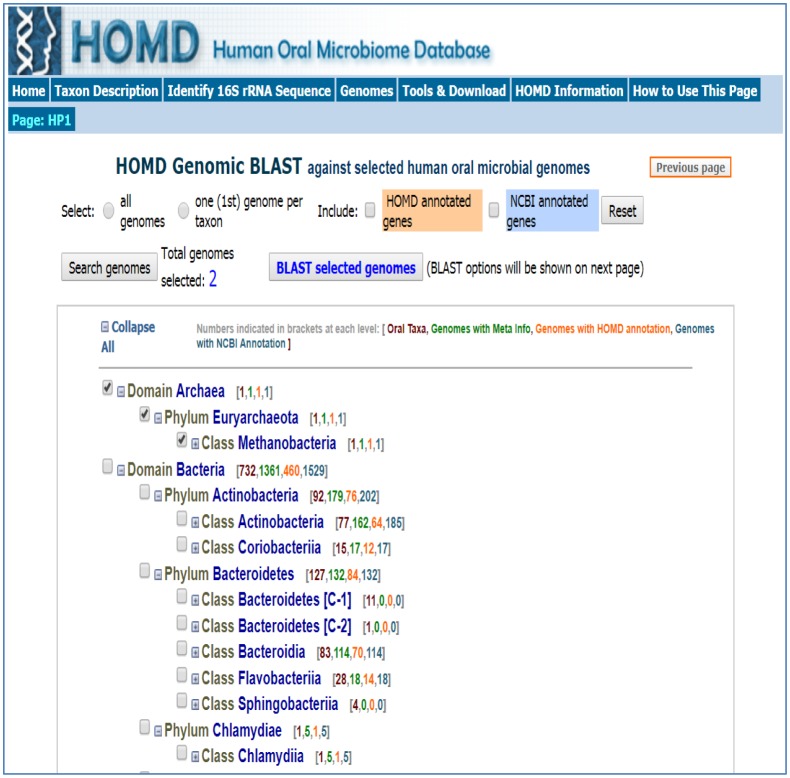
Human oral microbiome database [152].

Such approaches have helped in the development of database of all known oral bacterial species. Human Oral Microbiome database (HOMD), is a curated 16S rRNA database consisting of taxa that is presently known, deposited as oral bacterial genome sequences. This database also provides online tools for bacterial identification and characterization (Figure 2) [13],[152]. HOMD includes fifteen phyla of the bacterial domain of the oral origin namely *Actinobacteria, Bacteroidetes, Chlamydiae, Chlorobi, Chloroflexi, Euryarchaeota, Firmicutes, Fusobacteria, Gracilibacteria, Proteobacteria, Saccharibacteria, Spirochaetes, SR1, Synergistetes* and *WPS-2*. Majority of the above are oral commensals. Oral microbiome contains few archaebacteria mainly methanogens [65]. The oral cavity is one of the most well described microbiomes which cover a total of 392 taxa having one reference genome atleast and approaching around 1500 total genomes [153]. Fungal microbiota can be characterized using high throughput sequencing of Internal Transcribed Spacer 1 (ITS1) amplicon libraries [59]. Fungal mycobiome includes *Candida, Cladosporium, Alternaria, Aspergillus, Eurotium, Fusarium, Cryptococcus, Filobasidiella, Aureobasidium* and *Malassezia* [49]. Several *in vitro* and metagenomic studies reported that diverse kind of viruses and in particular bacteriophages inhabit the human body. For example, using NR BLASTX it has been found two types of phages. *Actinomyces* and *Streptococcus* phages are present in abundance [154]. Moreover with the use of advanced sequencing technologies based on CRISPR it has been formulated that these bacteriophages play a major role in modifying and deciding the bacterial communities of the oral cavity and also the diseases associated with them suggesting a complex interaction between bacteria and bacteriophages [50].

**Figure 3.**
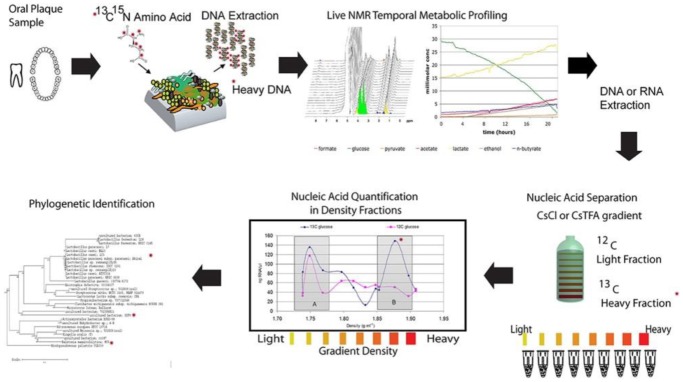
Illustration of a NMR-SIP experimental procedure [162].

With the advent of innovative techniques like Multiple Displacement Amplification (MDA) reaction, uncultivated microbes and divisions of bacterial phyla can be revealed and investigated. Single genome copy can be amplified for a more than billion times using sequencing from single bacterial cells through MDA reaction [155],[156]. Highly automated platform are recently being used for sequencing and assembly of single cell genomes of bacteria. In this technique single bacterial cells or small pools of cells are delivered into 384 well micro titer plates by Fluorescence Activated Cell Sorting (FACS). It is followed by automated cell lysis and DNA is further amplified by MDA method for the construction of genomic libraries derived from single cells, 16S rRNA gene amplification is performed for taxonomic profiling and diversity analysis. Amplified genomes can be further selected for whole genome sequencing. Genomes are then assembled using tools intended for MDA amplified single cells [157],[158]. This technique has increased the availability of the reference genomes for oral microbiome [159]. It is a tough task to determine that which microbes are executed for metabolizing substrates in a diverse microbial community. Metabolic networks of cultivated and uncultivated microbes in a complex biofilm can be identified using stable isotope probing (SIP) [160],[161]. In a study by Mclean et al. [162], functional microbial communities were identified using nucleic acid based SIP with *in situ* non-invasive Magnetic resonance spectroscopy (Figure 3). In this, real time organic acid production measurements were linked with active bacterial species in oral microbiome. The above analysis was carried out by incubating dental plaque samples from human subjects with isotopically labeled carbon sources such as 13C-Glucose in a defined minimal medium. NMR spectroscopy was performed for generating live metabolic profiles of the biofilms. This technique can be useful in identification of potential acid active species that can be responsible for enamel dissolution under a given set of conditions [163].

Human Oral Microbe Identification Microarray (HOMIM) is used in characterization of oral microbiome. This 16S rRNA based microarray contains more than 400 probes for the detection of variety of oral bacterial species. Oral disease such as progressive periodontitis including localized aggressive periodontitis (LAP) and generalized aggressive periodontitis (GAP) has been well studied by using HOMIM technology [164]. Effect of certain systemic diseases on the oral microbiome has also been analyzed using HOMIM. In pancreatic cancer patients salivary microbiota has been studied using HOMIM profiles. Sixteen bacterial species showed difference in pancreatic cancer samples and controls [165]. Advancements in next generation sequencing technologies has led to the up gradation of HOMIM platform by HOMINGS. It has a capability to identify approximately 600 oral bacterial taxa (http://homings.forsytyh.org/index2.html).

Microbial community functions in healthy and diseased conditions can be evaluated using high-throughput sequencing but comparing fluctuations in the metagenomics and meta transcriptones of dental plaques [166]–[168]. A study showed subgingival bacterial communities from periodontally healthy controls and subjects with chronic periodontitis demonstrated that microbial diversity is higher in case of individuals with disease than in healthy subjects. Diseased oral cavity showed presence of *Spirochaetes*, *Synergistetes*, *Bacteroidetes*, *Megasphaera*, *Parvimonas, Desulfobulbus, Clostridia*, *Negativicutes* and *Erysipelotrichia* [169]. Microbial diversity of dentine caries includes *Lactobacillus* *spp.*, *Prevotella* *spp.*, *Atopobium* *spp.*, *Olsenella* *spp.* and *Actinomyces spp.* [170]. Similar analysis in oral squamous cell carcinoma patients shows abundance of *Peptostreptococcus spp., Abiotrophia spp., Lactobacillus spp.* and *Micromonas spp*. in diseased condition [171]. Microbiomes associated with periodontal disease have a more diverse community structure which significantly resembles among different patients. Whereas, in healthy individual microbiome's taxanomic diversity is low but composition varies to a greater extent among individuals. Taxonomic data analysis shows that diseased microbiome undergoes a microbial community shift from being dominated by Gram-negative bacteria as compared to presence of Gram-positive bacteria in healthy microbiome [172].

Functional role of the microorganisms can be characterized at the community level by accessing its composition through metagenomics or analysis of gene transcripts by metatranscriptomics. First metagenomic study of oral microbiome was carried out by Xie et al. [173] by using next generation sequencing techniques. The metagenome of oral cavity under healthy and diseased state is described by Belda-Ferre et al. [166] while focussing on supragingival dental plaque and cavities. Eight samples with different health status were analysed using direct pyrosequencing. One Giga base pair of sequence was obtained which demonstrated the presence of complex bacterial communities in a dental cavity rather than sole dominance of *Streptococcus mutans*. Genes responsible for antimicrobial peptides and quorum sensing were present in the individuals who have not suffered from dental caries [166]. Metagenomic samples from dental plaques were analysed using different diversity indexes and rarefaction curves based on rRNA sequences which indicated presence of 73–120 genera. Lowest Common Ancestor (LCA) based approach can be used to classify metagenomic reads using databases to identify taxonomic groups [174].

Next generation sequencing generates large number of sequence reads which is leading into an era of meta-transcriptomics. Expressed transcripts referred to as RNA-seq can help to quantify novel transcripts. The transcriptomics study of oral bacterial communities are also employed in determining the potential differences in terms of gene expression between healthy and diseased conditions in dental caries, peridontol disease etc. [163],[175]. High-resolution RNA-seq analysis was used to propose *Fusobacterium nucleatum* as keystone species in periodontal disease. Lysine fermentation is the major metabolic pathway in the diseased condition as *F. nucleatum* is responsible for degradation of lysine into butyrate. Transcriptone based analysis in periodontal disease showed increased expression of butyrate production genes in *F. nucleatum*. This differential gene expression results promotion of the diseases [176]. RNA-seq analysis has helped in depth study of oral microbiome's metabolism. An approximate of 1100 enzyme encoding unique gene families have been recognized in the oral microbiome out of which 18% are expressed in a differential manner in diseased condition. Metabolic pathway database such as Kyoto Encyclopedia of Genes and Genomes (KEGG) has been employed in the analysis of differential gene expression. Studies show micobiomes in periodontal disease can produce intracellular toxins which can get accumulated in periodontal pockets due to reduced ability of their decomposition [154]. Enumeration of large number of species is possible through DNA-DNA hybridization. For bacteriome analysis of oral cavity 16S rRNA hypervariable regions V1 and V2 can be used to obtain species level taxonomic identities.

Analysis of proteins can also be undertaken by using techniques of metaproteomics. Combined action of the microbial community can also be detected by the analysis of final products originating from it [177]. Proteomics based studies can be useful in this regard as oral cavity consists of proteins from human and microbial origin. Microbiome colonizing on the oral surfaces contributes to the protein pool and hence these proteins can be indicative of the microorganisms present. Identification and quantification of metabolites in biological system is carried out through metabolomics. Oral metabolome finds its application in determination of biomarkers in oral cancers [178]. An elevated rate of degradation of macromolecules was shown in metabolome profiling in periodontal disease. Shifts in metabolic profiles indicate survival of pathogens in given conditions [179]. ORALGEN is a database that offers molecular information on oral pathogens. OralCard, is a web based application helping in the assessment and interpretation of oral proteome related data. Through its interactive data mining and associations, it can indicate involvement of the microbial protein in a pathological state [180].

Knowledge pool related to oral microbiome is enriched mainly with the findings of pure culture approaches. This is further upgraded by using culture independent methodology to classify uncultivated phylotypes and to understand microbial community level physiological relationships. Genome studies of complex microbial communities and their correlation with normal and diseased state of an individual is a futuristic approach providing vision about specific species, genes and their metabolism.

## 9. Conclusion

Biology of the oral cavity can be well understood from the information generated by high-throughput techniques followed by compilation and analysis of the data by using suitable bioinformatics tools. Due to complex variations among the members of oral microbiota, characterization at species level may not be enough to evaluate their pathogenic potential. Oral microbiome plays a key role in shaping up the host's health profile. Hence analysis of oral microbiome at an initial stage of chronic oral diseases such as periodontitis and dental caries would be helpful in earlier diagnosis and treatment of such diseases. The future approach should be based on analysis at community level in order to understand the various aspects of single species. There is an intricate relationship between oral microbiome and occurrence of other diseases like heart and liver related disorders, hence identification and characterization of causative organisms may act as an important gateway for maintenance of overall health. With latest evolution in fields of microbiomics, metagenomics and metatranscriptomics it has become at ease to relate complex oral microbial communities to various oral and non-oral conditions. Further the manipulation of oral microbiome by targeting specific species would pave a new hope for maintaining healthier oral community which will further help in maintenance of good health.
